# Work–Life Balance and Early Stage Careers: Dual Perspectives from One Household

**DOI:** 10.3389/fped.2015.00114

**Published:** 2015-12-22

**Authors:** Tristin Saravia, Jordy Saravia

**Affiliations:** ^1^Le Bonheur Children’s Hospital, Memphis, TN, USA; ^2^Department of Pediatrics, University of Tennessee Health Science Center, Memphis, TN, USA

**Keywords:** parenting, young professional, postdoc, work balance, life balance

We would like to preface this article by stating that it is written from a combined first-person perspective of both authors, with any person-specific text containing a respective name.

When one thinks about his/her general daily routine or how different aspects of the workplace interconnect with home life, there is likely no deeper self-inquiry into the actual formation of that routine; how is *this* my current norm, and what made it that way? In our early discussions of how we would share our perspective on “work–life balance,” we were faced with these types of questions. Fortunately for us, our answers are rooted in relatively recent times (past 3 years), so there is no doubt as to what has influenced our current lifestyle.

As a bit of background, we have been together for a total of 9 years and we just celebrated our third wedding anniversary. We both graduated with bachelor’s degrees from Louisiana State University and Jordy went on to pursue his PhD at LSU Health Science Center. During those graduate school years, we decided to move in together and Tristin was working very long hours managing two child and adolescent psychiatric clinics for a local physician. Then suddenly, life happened; we got married, found out we were expecting, and Jordy accepted a postdoctoral fellowship position in Tennessee – all within 3 months. In April 2013, we moved to Memphis where we had no family and few friends. There were some initial struggles securing a full-time job for Tristin, but this did allow her to spend 4 months with our son, Meyer, after he was born. Tristin eventually accepted a position as a residency coordinator at the University of Tennessee Health Science Center, and later moved on to a management position within the affiliated healthcare system. It was during this time of rapid change and adaptation that we were tasked with creating a balance between our careers and our home life.

Without a doubt, the most difficult and rewarding thing we have done is to rear our child completely on our own. Since we were located in a place without any nearby family to provide support and guidance, we had to learn how to independently function as a pair. Compared with our years in New Orleans, establishing a routine with two full-time jobs was much more difficult now that a child was involved. We had to adjust our morning regimen so that Meyer was always sufficiently taken care of by one of us while the other got ready; we had to allow ourselves more time (and coffee) to get him to daycare and get to work on time. If the baby was sick, we had to immediately evaluate one another’s work schedules to determine who goes in and who stays home – though we usually end up splitting the day, with one parent taking the morning shift and the other taking the afternoon. We are extremely dedicated to playing equal roles in our son’s life. One of us could easily handle feeding him dinner and putting him to bed every week night, but we make an effort to share those tasks for both ourselves and our child, as he is now at the stage when relationship satisfaction has significant influence on his long-term behavior and well-being (1, 2). We want him to rely on us equally and feel that he has a consistent, stable home environment with two available parents. There is no task that is specifically delegated to one of us, so that we are prepared to adapt to any situation that may suddenly arise. This is essential in establishing our current work–life balance because we focus on maximizing the time we spend together as a family, but we realistically understand each other’s circumstances with respect to work. Though our struggles to gain independence have not been easy, we are proud of what we have accomplished thus far and how it has undoubtedly strengthened our relationship.

As young professionals who are attempting to establish critical career foundations, we often sense an additional weight tipping the balance toward “work.” While we each maintain a focus for our respective jobs and goals, we also have genuine mutual respect for one another’s responsibilities. Managing multiple clinical offices/staff entails being readily available between 8:00 a.m. and 5:00 p.m. to manage/assist staff, troubleshooting issues that arise in the clinic, and participating in administrative meetings. Postdoctoral fellowships are said to be one of the most enjoyable stages of a scientist’s career because of an uninterrupted focus on doing science without the distractions of classes/exams like in graduate school nor the administrative/grant duties associated with being faculty. However, this “tween” status is a gray area in terms of salary and benefits (3), and the dwindling percentage of postdocs being hired as faculty has increased the pressure to work hard in this ultra-competitive field (4). In any case, the differential nature of our jobs has proven to be beneficial in ensuring that we each have adequate time for our work. A postdoc schedule is demanding, but the long hours and autonomy in the laboratory come with more flexibility compared to a more standardized “8-to-5” outpatient clinic schedule. If an experiment requires long incubation times or multiple time points, it can be timed as not to interfere with family time in the early evening; this typically results in returning to the laboratory later at night or early one morning on the weekend. Conversely, the slightly more rigid hours of an office manager is beneficial in that it creates a sense of structure to our day; we both know that around 5:00 p.m. it is time to start wrapping things up so we can go pick up the baby from daycare. Working in the same medical complex allows us the advantage of riding together to and from work. Using one vehicle (most days) further enforces a ~5:00 p.m. end to the work day, gives us extra time to interact with each other, and saves gas money. We both agree that seeing Meyer’s face light up when we walk into his classroom is the best part of the day, so riding together from work lets us both enjoy this bonus.

Our pledge to our son and to our careers would be unsustainable if we neglected the pledge that we made to each other. As difficult as it can be sometimes, we still set aside time and money to go on dates. These occasions serve as a needed distraction – an opportunity to experience something new to us in our new city, and a reminder of how much we enjoy each other’s company (and medium-rare steak). Alternatively, we enjoy simply being together and catching up on *Mad Men* or *Bob’s Burgers*. Having individual personal time is another vital part of our relationship. We have hobbies that we are separately invested in, and, more importantly, we understand how beneficial some degree of freedom is to the psyche (5). Supporting each other’s interests can be as simple as taking care of the baby alone for a few hours.

The changes in our work schedules since we moved to Memphis and became parents have been significant. Living in New Orleans, we sometimes did not eat dinner until 9:00 or 10:00 p.m. because we would wait for the other person to get home to enjoy it together. Weekends were reserved for Netflix binges or catching up on sleep in order to prepare for the upcoming week. Establishing this new balance, prompted by the birth of our child and life in a new city, has really encouraged us to enjoy life more for ourselves and Meyer. We acknowledge that these are the formative years of our careers, but importantly these are also the formative years of our family and ensuring that Meyer has a loving, healthy upbringing. Additionally, we have learned to depend on one another and view outside help as a luxury rather than a necessity. Knowing how much we can rely on one another, how much we support each other’s career goals, and how much we value our relationship has truly brought us closer together as a family.

Though we are content with our current dynamic, we acknowledge that things will certainly change in coming years. As our careers continue to advance, we will be faced with increasing work responsibility that will undoubtedly put more strain on achieving a good work–life balance. We are fully aware of the potential negative ramifications looming in the future, should we fail in our attempt to maintain balance. Despite knowing that “winter is coming,” we uninspiringly concede that we do not yet specifically know how we will adapt in the future. What comforts us about that fact is that, in retrospect, we felt the exact same way 3 years ago when looking forward to our current situation. Based on experiences leading up until now, our opinion is that a positive work–life balance is attainable if we continue to approach new situations in the ways that have worked for us thus far – truly open communication, unconditional support, equality, flexibility, and utilization of complementary strengths (and medium-rare steak).

## Author Contributions

Both authors equally contributed to the conception and writing of this work.

## Conflict of Interest Statement

The authors declare that the research was conducted in the absence of any commercial or financial relationships that could be construed as a potential conflict of interest.
